# The profiles and clinical significance of extraocular muscle-expressed lncRNAs and mRNAs in oculomotor nerve palsy

**DOI:** 10.3389/fnmol.2023.1293344

**Published:** 2023-12-20

**Authors:** Lianqun Wu, Mingsu Shi, Yu Liang, Jiaqiu Huang, Weiyi Xia, Hewei Bian, Qiao Zhuo, Chen Zhao

**Affiliations:** ^1^Eye Institute and Department of Ophthalmology, Eye and ENT Hospital, Fudan University, Shanghai, China; ^2^NHC Key Laboratory of Myopia (Fudan University), Key Laboratory of Myopia, Chinese Academy of Medical Sciences, Shanghai, China; ^3^Shanghai Key Laboratory of Visual Impairment and Restoration, Shanghai, China

**Keywords:** long non-coding RNA, mRNA, extraocular muscle, oculomotor nerve palsy, constant exotropia

## Abstract

**Introduction:**

Oculomotor nerve palsy (ONP) arises from primary abnormalities in the central neural pathways that control the extraocular muscles (EOMs). Long non-coding RNAs (lncRNAs) have been found to be involved in the pathogenesis of various neuroparalytic diseases. However, little is known about the role of lncRNAs in ONP.

**Methods:**

We collected medial rectus muscle tissue from ONP and constant exotropia (CXT) patients during strabismus surgeries for RNA sequencing analysis. Differentially expressed mRNAs and lncRNAs were revealed and included in the functional enrichment analysis. Co-expression analysis was conducted between these differentially expressed mRNAs and lncRNAs, followed by target gene prediction of differentially expressed lncRNAs. In addition, lncRNA-microRNA and lncRNA-transcription factor-mRNA interaction networks were constructed to further elaborate the pathological changes in medial rectus muscle of ONP. Furthermore, RT-qPCR was applied to further validate the expression levels of important lncRNAs and mRNAs, whose clinical significance was examined by receiver operating characteristic (ROC) curve analysis.

**Results:**

A total of 618 differentially expressed lncRNAs and 322 differentially expressed mRNAs were identified. The up-regulated mRNAs were significantly related to cholinergic synaptic transmission (such as *CHRM3* and *CHRND*) and the components and metabolism of extracellular matrix (such as *CHI3L1* and *COL19A1*), while the down-regulated mRNAs were significantly correlated with the composition (such as *MYH7* and *MYL3*) and contraction force (such as *MYH7* and *TNNT1*) of muscle fibers. Co-expression analysis and target gene prediction revealed the strong correlation between *MYH7* and *NR_126491.1* as well as *MYOD1* and *ENST00000524479*. Moreover, the differential expressions of lncRNAs (*XR_001739409.1*, *NR_024160.1* and *XR_001738373.1*) and mRNAs (*CDKN1A*, *MYOG*, *MYOD1*, *MYBPH*, *TMEM64*, *STATH*, and *MYL3*) were validated by RT-qPCR. ROC curve analysis showed that lncRNAs (*XR_001739409.1*, *NR_024160.1*, and *NR_002766.2*) and mRNAs (*CDKN1A*, *MYOG*, *MYOD1*, *MYBPH*, *TMEM64*, and *STATH*) might be promising biomarkers of ONP.

**Conclusions:**

These results may shed light on the molecular biology of EOMs of ONP, as well as the possible correlation of lncRNAs and mRNAs with clinical practice.

## 1 Introduction

Oculomotor nerve palsy (ONP), also known as third cranial nerve palsy, is a common and important clinical entity, which may be congenital or acquired. The most common causes of acquired ONP involve vascular related etiologies such as aneurysm, followed by idiopathic causes, trauma, and neoplasm (Kim et al., 2018; Raza et al., 2018). The symptoms can range from a complete palsy, including mydriasis, ptosis, and a ‘down and out’ appearance of the affected eye, to a milder form involving any combination of affected muscles and severity at any level (Pineles and Velez, 2018).

ONP arises from primary abnormalities in the central neural pathways that control the extraocular muscles (EOMs) (Tychsen, 2005; Das and Mustari, 2007), but the surgical management of ONP patients is at the level of the EOMs, which depends on the number of EOMs involved and the severity of rotation deficiency (Sadagopan and Wasserman, 2013). Although appropriate surgical techniques can be rewarding for ONP patients (Singh et al., 2016; Nelson, 2019), no statistically significant differences have been found between the surgical options and long-term aesthetic and functional outcomes (Merino et al., 2019), which makes the management of ONP a great challenge even for the most experienced surgeon. Thus, further insight into the pathological characteristics of ONP is urgently needed for better treatment.

EOMs, composed of four recti muscles, two oblique muscles and a levator palpebral muscle, are a group of specialized muscles that are responsible for locating and accurately tracking objects by the visual system (Fischer et al., 2005). Studies have revealed the differences in genetic characteristics between strabismic and normal EOMs, with up-regulation in genes involved in extracellular matrix structure, and down-regulation in genes related to contractility (Altick et al., 2012; Agarwal et al., 2016). However, the investigation on the molecular biology of EOMs is little known. Furthermore, denervation-induced alterations in gene expression have been found in skeletal muscles (Magnusson et al., 2005; Shen et al., 2019), but the effect of ONP on the molecular features of EOMs remains unrevealed.

Long non-coding RNAs (lncRNAs) refer to a diverse class of non-coding transcripts with a length of more than 200 nucleotides that do not encode protein. Through an increasing number of sequencing-based approaches, the structures and interactions of lncRNAs have been revealed in the transcriptomes of multiple species (Qian et al., 2019). Studies have indicated that lncRNAs may play a critical role in the regulation of gene expression and that their dysfunction may have impact in several pathologies, including cancer and immune responses (Fernandes et al., 2019; Statello et al., 2021). Moreover, lncRNAs have been found to be linked to the pathogenesis and prognosis of several neuroparalytic diseases, such as progressive supranuclear palsy (Jabbari et al., 2021), diabetic peripheral neuropathy (Wang et al., 2020) and thyrotoxic periodic paralysis (Melo et al., 2017). However, the lncRNA expression profile in EOMs of ONP remains unknown.

In this study, we collected medial rectus muscle tissue from ONP and constant exotropia (CXT) patients during strabismus surgeries for RNA sequencing analysis, as it is the affected and only accessible EOM during surgery for ONP patients. Differentially expressed mRNAs and lncRNAs were revealed and included in the functional enrichment analysis. Co-expression analysis was conducted between these differentially expressed mRNAs and lncRNAs, followed by target gene prediction of differentially expressed lncRNAs. In addition, lncRNA-micro RNA (miRNA) and lncRNA-transcription factor (TF)-mRNA interaction networks were constructed to further elaborate the pathological changes in the EOMs of ONP. Furthermore, real-time quantitative polymerase chain reaction (RT-qPCR) was applied to further validate the expression levels of important lncRNAs and mRNAs, whose clinical significance was examined by receiver operating characteristic (ROC) curve analysis.

## 2 Materials and methods

### 2.1 Study population and tissue sample

We recruited patients diagnosed with congenital or acquired ONP from September 2019 to February 2022 at the Eye and Ear, Nose, and Throat (EENT) Hospital, Fudan University, China. All patients had a duration of symptoms for more than 6 months, and underwent surgery for lateral rectus recession and medial rectus strengthening, with or without vertical offsets, by a single surgeon (C. Z). Patients with any other cranial nerve palsy, any other disease involving the EOMs, previous strabismus surgery or follow-up <3 months were excluded. Aside from typical clinical presentation, congenital ONP was differentiated from congenital fibrosis of the extraocular muscles type 3 (CFEOM3), which is mainly caused by mutations in the *TUBB3* gene (Poirier et al., 2010). Sanger sequencing was applied to exclude the mutation at c.785G > A (p.Arg262His) site (Supplementary Figure 1; Whitman et al., 2021). A total of 22 ONP patients were recruited for our study and 5 of them were subsequently excluded due to their failure of quality inspection. The clinical information of the 17 ONP patients included in our study is summarized in Table 1.

**TABLE 1:** Clinical information of oculomotor nerve palsy patients in this study.

No	Age (year)	Sex	Etiology	Duration (year)	Affected eye	Ptosis	Exodeviation (PD)	Vertical deviation (PD)	Degree of adduction deficits	The amount of MR resection (mm)
1	46	Female	Trauma	0.92	Right	No	90	RHT20	−3	10
2	30	Female	Meningitis	25	Right	No	100	RHT15	−4	8
3	26	Male	Tumor	9	Both	No	100		−3	7
4	23	Male	Tumor	2	Left	Left	60		−2	7
5	64	Female	Hemangioma	2.75	Left	No	85		−2	9
6	38	Female	Unknown	35	Right	No	100	RHT30	−3	11
7	23	Female	Hemangioma	1	Right	No	90	RHT25	−2	9
8	5	Male	Congenital	5	Right	Right	35	LHT10	−2	7
9	5	Female	Congenital	5	Left	Left	80		−2	11
10	15	Female	Unknown	8	Left	Left	100		−2	12
11	5	Male	Hemangioma	1.08	Both	No	100		−2	11
12	5	Male	Trauma	0.92	Left	Left	70		−1	9.5
13	1	Male	Congenital	1	Left	No	>100		−2	11
14	7	Female	Congenital	7	Left	No	76		−1	9.5
15	12	Female	Unknown	5	Right	No	35	RHT5	−5	9.5
16	28	Male	Congenital	28	Left	No	40		−2	5
17	6	Female	Congenital	6	Left	Left	70		−4	11

PD, prism diopter; MR, medial rectus; RHT, right hypertropia; LHT, left hypertropia.

Patient No. 1 to No. 4 were included in RNA sequencing, and Patient No. 1 to No. 16 were included in RT-qPCR validation. Patient No. 17 was used for histopathologic analysis.

Patients diagnosed with CXT were enrolled as controls, including infantile constant exotropia as well as intermittent exotropia presenting as a constant pattern. All of them underwent the same surgical procedures as the ONP group during the same study period by the same surgeon. Patients with a secondary cause of strabismus, a history of strabismus surgery or follow-up <3 months were excluded. The CXT patients used as controls for RNA sequencing were matched for age and gender. For patients included in RT-qPCR, no significant difference was found in age (Mann-Whitney test, *p* = 0.786) and gender (Chi-square test, *p* = 0.930) between ONP and CXT groups. A total of 16 CXT patients were recruited for our study and 5 of them were subsequently excluded. The clinical information of the 11 CXT patients included in our study is summarized in Table 2.

**TABLE 2:** Clinical information of constant exotropia patients in this study.

No	Age (year)	Sex	Duration (year)	Exodeviation (PD)	Vertical deviation (PD)	The amount of MR resection (mm)
1	19	Female	19	60	RHT10	5
2	38	Female	10	90		5.5
3	44	Male	44	>110		5
4	21	Male	21	64		5
5	10	Female	6	76		5
6	29	Female	29	100	LHT5	6
7	9	Male	7	66		5
8	11	Male	1	72		5
9	9	Male	4	70		5
10	21	Female	21	68		5
11	11	Female	3	74		5

PD, prism diopter; MR, medial rectus; RHT, right hypertropia; LHT, left hypertropia.

Patient No. 1 to No. 4 were included in RNA sequencing, and Patient No. 1 to No. 10 were included in RT-qPCR validation. Patient No. 11 was used for histopathologic analysis.

All patients have gone through a complete ocular examination, as well as sensory and motor evaluation, as previously published (Wu et al., 2020a). In brief, a prism alternating cover test (PACT) was applied to measure the horizontal and vertical deviations. For the paretic eyes and patients who were incapable of cooperating with the PACT, a Krimsky test was applied. Duction deficits were measured on a −5 to 0 scale, with −5 indicating inability of the eye to achieve midline; −4, ability to just reach midline; −3, ability to cross midline but 75% deficit left; −2, ability to cross midline but 50% deficit left; −1, ability to cross midline but 25% deficit left (Holmes et al., 2001; Hatt et al., 2017).

For the ONP patients included in this study, a recess–resect procedure was required to correct the large-angle exotropia, thereby making medial rectus muscle the only EOM available during surgery. Medial rectus muscle tissue samples were derived from the wastes during strabismus surgery of both the ONP group and CXT group, and were immediately stored in a −80°C freezer.

### 2.2 RNA extraction and sequencing analysis

Medial rectus muscle tissue samples from four ONP patients and four CXT patients were harvested into TRIzol reagent (Invitrogen, Carlsbad, CA, USA). Total RNAs were isolated based on the recommended manufacturer’s protocols. Nanodrop 2000 (Thermo Scientific, Wilmington, DE, USA) was applied to measure the quality and quantity of the total RNA samples, while the integrity was evaluated on an Agilent 2100 Bioanalyzer (Agilent Technologies, Santa Clara, CA, USA). Samples with an RNA integrity number (RIN) ⩾ 8 were applied for subsequent analysis (Wu et al., 2020b).

Afterwards, we removed ribosomal RNA (rRNA) from the extracted total RNA, and strand-specific RNA-seq libraries were constructed by TruSeq Stranded Total RNA with Ribo-Zero Gold (Illumina, San Diego, CA, USA) following the manufacturer’s protocols. An Agilent 2100 Bioanalyzer (Agilent Technologies) was applied for library quality control. Raw reads of fastq format were firstly processed using fastp (Chen et al., 2018) and the low-quality reads were removed to obtain the clean reads. The proportion of valid bases exceeded 85% in all samples. High-throughput RNA sequencing was conducted on the Illumina NovaSeq 6000 System (Illumina) by performing a paired-end run with a 150 bp read length (Wu et al., 2021).

### 2.3 LncRNA prediction, annotation, and quantification

Based on the results of the genome comparison of different samples, Stringtie software was used to merge the constructed transcripts (Pertea et al., 2015). The merged transcripts were compared with the reference transcripts one by one using cuffcompare software, and known coding transcripts or new transcripts of known loci were screened. The reference genome for sequence alignment was from Genome Database (version GRCh38.p12). The transcripts were further screened according to the length > 200 nt and number of exons ≥ 2. CPC (Kang et al., 2017), CNCI (Sun et al., 2013), Pfam (Sonnhammer et al., 1998), and PLEK (Li et al., 2014) were applied to screen out transcripts with coding potential. Finally, the transcripts were compared and merged with known lncRNAs using blastn software.

The sequencing reads of each sample were compared with reference transcripts to calculate the expression abundance using bowtie2 (Langmead and Salzberg, 2012) and eXpress (Roberts and Pachter, 2013). Fragments per KB per million reads (FPKM) method was used on the basis of the following formula (Roberts et al., 2011):


$$\begin{array}{l} F\,P\,K\,M\\ =\frac{C\,o\,u\,n\,t\,s\,o\,f\,m\,a\,p\,p\,e\,d\,f\,r\,a\,g\,m\,e\,n\,t\,s}{T\,o\,t\,a\,l\,C\,o\,u\,n\,t\,o\,f\,m\,a\,p\,p\,e\,d\,f\,r\,a\,g\,m\,e\,n\,t\,s\,L\,e\,n\,g\,t\,h\,o\,f\,t\,r\,a\,n\,s\,c\,r\,i\,p\,t}\times 10^{9} \end{array}$$

### 2.4 Differential expression analysis and functional analysis

The DESeq package (Anders and Huber, 2012) in R was used to screen out differentially expressed lncRNAs and mRNAs, with a cut-off criteria of *p*-value < 0.05 and |log2 Fold Change (FC)| > 1. Variance-mean dependence in the count data from RNA sequencing was estimated using the R package of DESeq, as well as tests for differential expression on the basis of a model using the negative binomial distribution.

Gene ontology (GO) functional enrichment analysis^1^ was applied for differentially expressed lncRNAs and mRNAs in the categories of biological process (BP), cellular component (CC) and molecular function (MF). Top 30 GO entries were screened on the basis of a descending order of -log10 *p*-value in each category, with differential genes > 2.

In addition, pathway enrichment analysis was performed for differentially expressed lncRNAs and mRNAs according to the Kyoto encyclopedia of genes and genomes (KEGG) database^2^ (Kanehisa et al., 2008). The top 20 KEGG entries were screened according to the descending order of -log10 *p*-value, with the number of differential genes > 2.

### 2.5 LncRNA-mRNA co-expression analysis and lncRNA target gene prediction

The correlation of differentially expressed lncRNAs with mRNAs was assessed according to their expression level using Pearson’s correlation test. The top 500 differentially expressed lncRNAs based on their |log2 FC| with a length < 6,000 nt were included in the analysis. Co-expression pairs with a *p*-value ⩽ 0.05 and Pearson’s correlation coefficient ⩾ 0.8 were screened and visualized with a circos plot (Krzywinski et al., 2009).

Then, FEELnc software (Wucher et al., 2017) was used to screen out all coding genes in 100k upstream or downstream of the differentially expressed lncRNAs, which were intersected with the genes significantly co-expressed with differentially expressed lncRNAs according to Pearson’s correlation test. The obtained genes were considered to be *cis* regulated by differentially expressed lncRNAs.

In addition, RNA interaction software RIsearch (version 2.0) (Alkan et al., 2017) was applied to predict the binding potential of co-expressed lncRNAs and mRNAs, with a cut-off criterion of interacted base length ≥ 10 and binding free energy ≤ −100 Kcal/mol. The top 40 correlated pairs according to the co-expression *p*-value were applied for the construction of the lncRNA-target regulatory network, which was visualized by the network software package (Butts, 2008).

### 2.6 Construction of lncRNA-miRNA and lncRNA-TF-mRNA network

Since there are multiple miRNA binding sites on lncRNAs (Quinn and Chang, 2016), we applied the method of miRNA target gene prediction to identify lncRNAs binding to miRNAs. Interactions between lncRNAs and miRNAs were predicted using miranda software (John et al., 2004) with a threshold of *p*-value < 0.05. The top 300 correlated lncRNA-miRNA pairs based on the *p*-value of interaction were applied for the construction of lncRNA-miRNA interaction network. The diagram was drawn using an R network package.^3^

The top 20 differentially expressed lncRNAs by *cis* regulation and the top 20 co-expressed mRNAs of the lncRNAs based on the *p*-value of interaction were included in the construction of the lncRNA-TF-mRNA network. According to the data from JASPAR 2020 (Fornes et al., 2020), we predicted the potential binding TFs of lncRNAs. We then adopted the gene-TF relationship provided by GTRD database^4^ (Yevshin et al., 2017) and the result of lncRNA-mRNA co-expression analysis to construct the lncRNA-TF-mRNA network.

### 2.7 RT-qPCR

In order to further verify the results of the RNA sequencing data, the expression levels of several important lncRNAs and mRNAs were detected by RT-qPCR. First, we isolated total RNA from the medial rectus muscle tissue samples from 16 ONP patients and 10 CXT patients using TRIzol reagent (Invitrogen). Then, the cDNA was synthesized from 500 ng of extracted total RNA using the TransScript All-in-One First-Strand cDNA Synthesis SuperMIX for qPCR Kit (TransGen Biotech, Beijing, China) and amplified by RT-qPCR with a PerfectStartTM Green qPCR SuperMix Kit (TransGen Biotech) on a LightCycler^®^ 480 II Real-time PCR instrument (Roche, Basel, Switzerland). The housekeeping gene glyceraldehyde-3-phosphate dehydrogenase (GAPDH) was applied as an internal control. The 2-^ΔΔCt^ method was adopted to quantify the relative quantification of gene expression levels (Livak and Schmittgen, 2001). FastPCR (Kalendar, 2022) was used to calculate primer efficiency. The sequences and primer efficiency of the primers are shown in Table 3.

**TABLE 3:** Primer sequences for RT-qPCR analysis.

Name	Forward primer (5– >3)	Primer efficiency (%)	Reverse primer (5– >3)	Primer efficiency (%)	Product length (bp)
GAPDH	CCTCACAGTTGCCATGTAGA	81%	TGGTACATGACAAGGTGCG	91%	69
CDKN1A	CGACTGTGATGCGCTAAT	83%	GTGGTGTCTCGGTGACAA	86%	80
MYOG	CGTGTAAGGTGTGTAAGAGGA	75%	CCTCATTCACCTTCTTGAGC	82%	93
MT1X	GACAGCTGTGCTCTCAGA	85%	AAGATGTAGCAAACGGGTC	83%	97
MYOD1	GCCTTTGAGACACTCAAGC	71%	TCGATATAGCGGATGGCG	100%	92
MYBPH	AGGACTCCATCCTCTTCATTC	74%	GGATGTCAATGACTGCCTT	76%	105
TMEM64	CAGACTGACACCCATACCT	85%	CGAAGATGCCATCAGATAGTTG	64%	88
STATH	AAGTTCCTTGTCTTTGCCT	77%	GGGCCATACCCATACTCT	64%	83
MYL3	CCCAAGAAGGATGATGCCA	85%	GCATCAAACTCGACCTCCTTA	82%	95
MYH7	AGATGGAAGGAGACCTCAAT	78%	CTCTGGAGGCTCTTGACT	86%	94
TNNT1	GTCAGAGAGAGCCGAGCAA	98%	CTTCCTCTTCCTTCCTCATC	77%	95
XR_001750763.2	TACCACCTAGTACAGTGCCT	79%	AGAACTCTCAGAGTCATCCTT	67%	96
XR_001739409.1	CAAGCCAGGACATGCTACA	78%	TAGCAGCAAGGCACTTAGAG	73%	89
NR_024160.1	TGCTTGAAAGGAGGTAGTAGTC	71%	GCATCACATGCTCATGGTAA	80%	112
NR_034011.1	GGATTAGGTGAAAGAAGCTGAG	70%	TAAACTTCGCCCATTACCACT	81%	93
NR_022008.1	AGAGTGTGTTCTATGTTCACTG	67%	CTGAGACAAGAAGTGCTTGAAT	69%	101
ENST00000566098	AAATGGTTAAGATGGCTGGG	75%	AGGCAGGAGAATCACTTGA	71%	85
NR_002766.2	CTGGATCCCACCAACATACA	76%	GTCAGTTCCGGTCCTCTT	93%	82
NR_125872.1	ACCAGCTTCAGATACATGC	76%	TGGCGTAGTTATCATCCACA	66%	102
NR_126491.1	TGAATCTGGGTTACCTTCAACA	63%	GGTGAAGAAGAAGATGGAAGGA	71%	129
XR_001738373.1	TCCCTCTCCTCTCTTAGCC	85%	TCTTCCCATGCAGCAGAT	88%	91
ENST00000421842	TCTGATGGATTTGTTGACTCCT	71%	GAAACTGACGTCCACAGC	96%	93

### 2.8 Histopathologic analysis

Tissue samples were fixed in 4% polymethylaldehyde solution for 24 h. After alcohol gradient dehydration and clearing in xylene, the samples were embedded in paraffin and sliced into 4 μm thick sections.

Hematoxylin/eosin (HE) staining was conducted according to routine protocols. In brief, after deparaffinization and rehydration, tissue sections were stained with hematoxylin solution (Ribiology, Shanghai, China) for 5 min followed by several dips in 1% acid ethanol and then rinsed in distilled water. Then the sections were stained with eosin solution (Ribiology) for 2 min. Masson staining was conducted using Masson staining kit (Ribiology) according to the manufacturer’s protocol. After staining, the sections were dehydrated with graded alcohol and cleared in xylene. Images were captured with Axio Observer 3 (Carl Zeiss, Oberkochen, Germany) using ZEN software (Carl Zeiss).

### 2.9 Statistical analysis

Statistical analyses were conducted based on GraphPad Prism (Version 9.00). A Student’s *t*-test was applied to check the difference in lncRNA and mRNA levels between the ONP and CXT groups. A significant difference was established when *p* < 0.05. The ROC curve and the area under the curve (AUC) were used to evaluate the diagnostic value of lncRNAs and mRNAs.

## 3 Results

### 3.1 Pathological changes in the EOMs of ONP

Hematoxylin/eosin and Masson staining was applied to reveal the pathological changes in the EOMs of ONP. As a result, atrophy and fibrosis of muscle fibers was seen in the EOMs of ONP (Supplementary Figure 2), which is a common pathological change of denervated muscle.

### 3.2 Distribution and location of novel lncRNAs

**FIGURE 1:**
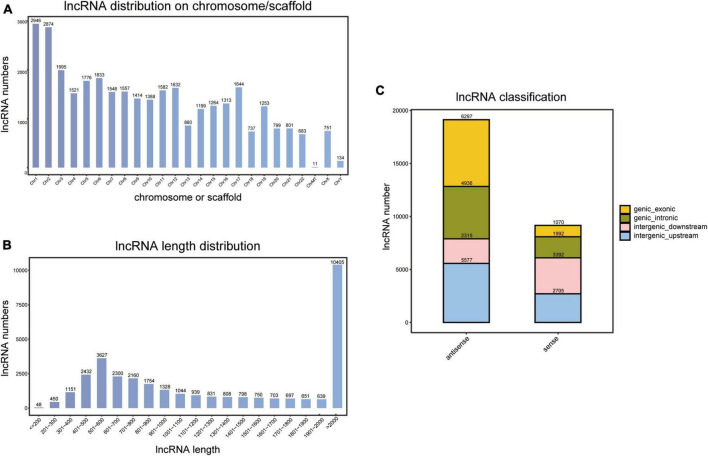
Identification of novel long non-coding RNAs (lncRNAs). **(A)** Chromosome or scaffold distribution of novel lncRNAs. **(B)** Length distribution of novel lncRNAs. **(C)** Classification of novel lncRNAs.

As indicated in Figure 1A, novel lncRNAs are encoded on all chromosomes, including ChrMT (mitochondrial) and ChrY. However, the number and distribution of encoded regions greatly differ across different chromosomes. Chr1 (8.8%, 2,946/33,515) and Chr2 (8.6%, 2,874/33,515) are the chromosomes with a large number of encoded lncRNA genes and high transcriptional activity, whereas ChrMT (0.033%, 11/33,515) and ChrY (0.40%, 134/33,515) contain very few lncRNA genes, and have a comparatively low transcriptional activity.

Moreover, length distribution indicated that lncRNAs with a length of more than 2,000 nt accounted for the largest proportion (31.0%, 10,405/33,515), and 10.8% (3,627/33,515) had a length of 501–600 nt (Figure 1B).

By comparing to transcripts of coding proteins, we calculated the location of novel lncRNAs. As a result, 49.5% (13,989/28,284) of lncRNAs were from intergenic regions, 24.5% (6,928/28,284) were from intronic regions, and 26.0% (7,367/28,284) were from exonic regions (Figure 1C).

### 3.3 Expression profiles of lncRNAs and mRNAs

**FIGURE 2:**
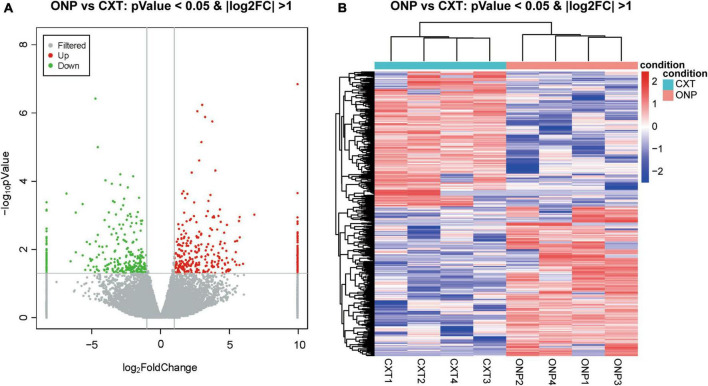
LncRNA sequencing analysis of the differential expression between oculomotor nerve palsy (ONP) and constant exotropia (CXT) groups. **(A)** Volcano plot displaying the differential lncRNA expression between ONP and CXT patients; red and green dots represent up-regulation and down-regulation, respectively, while gray dots indicate no significant change. **(B)** Hierarchical clustering heatmap demonstrating the differential lncRNA expression between medial rectus muscle samples of ONP and CXT groups; red and blue dots indicate up-regulation and down-regulation, respectively.

A total of 618 differentially expressed lncRNAs were screened, composed of 325 up-regulated and 293 down-regulated lncRNAs (Figure 2A). Based on a hierarchical clustering heatmap, we found that the expression patterns of these differentially expressed lncRNAs were significantly different between ONP and CXT groups (Figure 2B).

**FIGURE 3:**
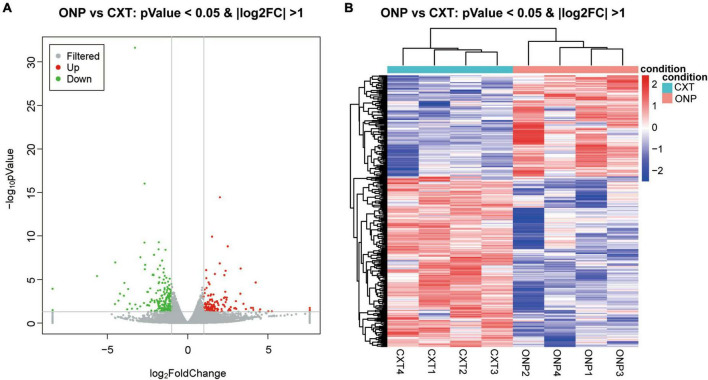
MRNA sequencing analysis of the differential expression between medial rectus muscle samples from ONP and CXT patients. **(A)** Volcano plot displaying the differential mRNA expression between ONP and CXT patients; red and green dots represent up-regulation and down-regulation, respectively, while gray dots indicate no significant change. **(B)** Hierarchical clustering heatmap demonstrating the differential mRNA expression between medial rectus muscle samples of ONP and CXT groups; red and blue dots indicate up-regulation and down-regulation, respectively.

With regard to mRNAs, 120 up-regulated and 202 down-regulated mRNAs were identified (Figure 3A). Heatmap shows the hierarchical clustering of these differentially expressed mRNAs (Figure 3B).

### 3.4 GO and KEGG analyses of differentially expressed lncRNAs

**FIGURE 4:**
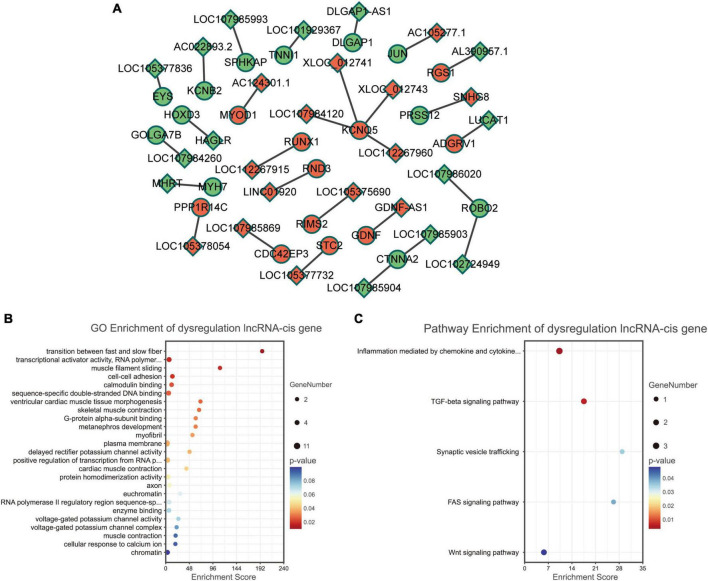
Functional enrichment analyses for differentially expressed lncRNAs. **(A)** Dysregulated lncRNA-*cis* gene network. Circles and diamonds indicate genes and lncRNAs, respectively. Red and green represent up-regulation and down-regulation, respectively. **(B)** Gene Ontology (GO) analysis of dysregulated lncRNA-*cis* genes. **(C)** Kyoto encyclopedia of genes and genomes (KEGG) analysis of dysregulated lncRNA-*cis* genes.

We analyzed the differentially expressed genes which are *cis-*regulated by the dysregulated lncRNAs. As a result, only 23 genes *cis-*regulated by 28 dysregulated lncRNAs were differentially expressed (Figure 4A).

According to GO functional enrichment analysis, the dysregulated lncRNAs were mainly involved in the contraction force of muscle fibers (such as GO terms “transition between fast and slow fiber” and “muscle filament sliding”) and regulation of transcription (such as GO terms “transcriptional activator activity” and “RNA polymerase II transcription regulatory region sequence-specific binding”) (Figure 4B).

Kyoto encyclopedia of genes and genomes pathway enrichment analysis revealed that the dysregulated lncRNAs were significantly correlated with some immune and inflammatory processes (such as KEGG terms “inflammation mediated by chemokine and cytokine signaling pathway” and “TGF-beta signaling pathway”) (Figure 4C).

### 3.5 Functional enrichment analysis of differentially expressed mRNAs

**FIGURE 5:**
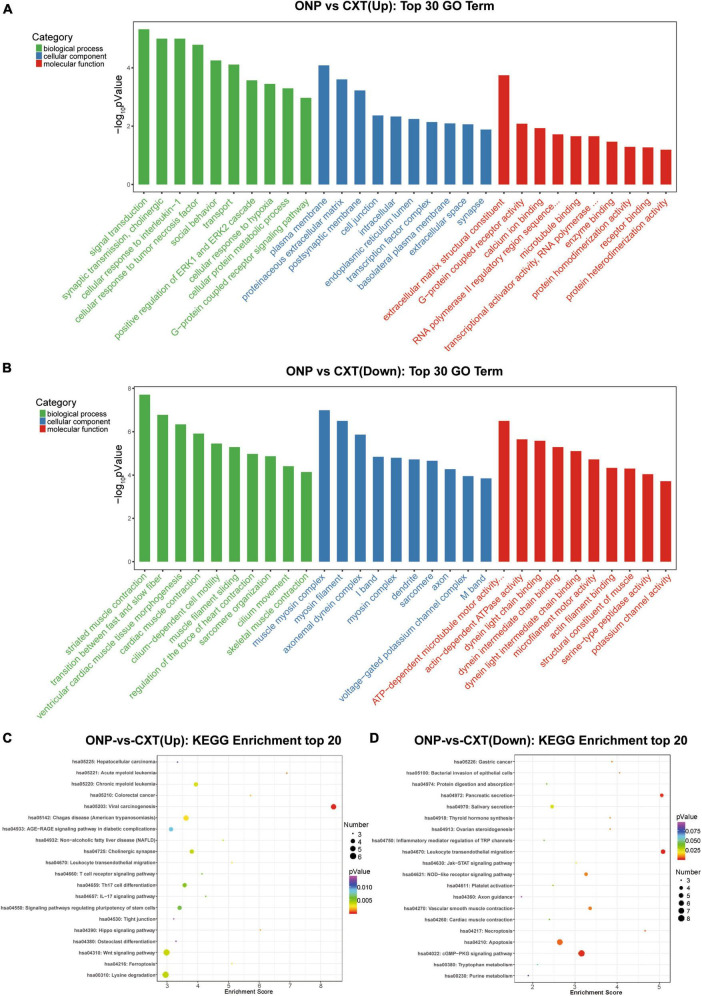
Functional enrichment analyses for differentially expressed mRNAs. Top 30 GO terms in the biological process (BP), cellular component (CC) and molecular function (MF) categories enriched for **(A)** up-regulated mRNAs and **(B)** down-regulated mRNAs. Top 20 KEGG pathways enriched for **(C)** up-regulated mRNAs and **(D)** down-regulated mRNAs.

According to GO analysis, the up-regulated mRNAs mainly functioned in synaptic transmission, cholinergic (such as *CHRM3* and *CHRND*) in the BP category; proteinaceous extracellular matrix (such as *CHI3L1*, *IL1RL1*, *COL19A1*, *TECTA*, *SPON1*, and *LRFN2*) in the CC category; and extracellular matrix structural constituent (such as *CHI3L1*, *COL19A1*, and *TECTA*) in the MF category (Figure 5A). However, down-regulated mRNAs mainly functioned in striated muscle contraction (such as *MYH7*, *MYBPHL*, *MYH6*, *MYOM2*, and *MYLK2*) and the transition between fast and slow fibers (such as *MYH7*, *TNNT1*, and *TNNI1*) in the BP category; muscle myosin complex (such as *MYH7*, *MYL3*, *MYBPHL*, *MYH6*, and *MYOM2*) and myosin filament (such as *MYH7*, *MYH6*, and *MYOM2*) in the CC category; and actin-dependent ATPase activity (such as *MYH7* and *MYH6*) and dynein light chain binding (such as *DNAH8* and *DNAH3*) in the MF category (Figure 5B).

In addition, as a result of KEGG analysis, up-regulated mRNAs were significantly related to calcium signaling pathway (such as *CHRM3* and *CACNA1I*) and neuroactive ligand-receptor interaction (such as *CHRM3* and *CHRND*) (Figure 5C). Down-regulated mRNAs were significantly correlated with neuroactive ligand-receptor interaction (such as *GABRB1* and *GABRB2*), calcium signaling pathway (such as *NOS1* and *MYLK3*) and regulation of actin cytoskeleton (such as *FGF6*, *MYLK3*, and *MYLK2*) (Figure 5D).

### 3.6 Co-expression analysis and target gene prediction

**FIGURE 6:**
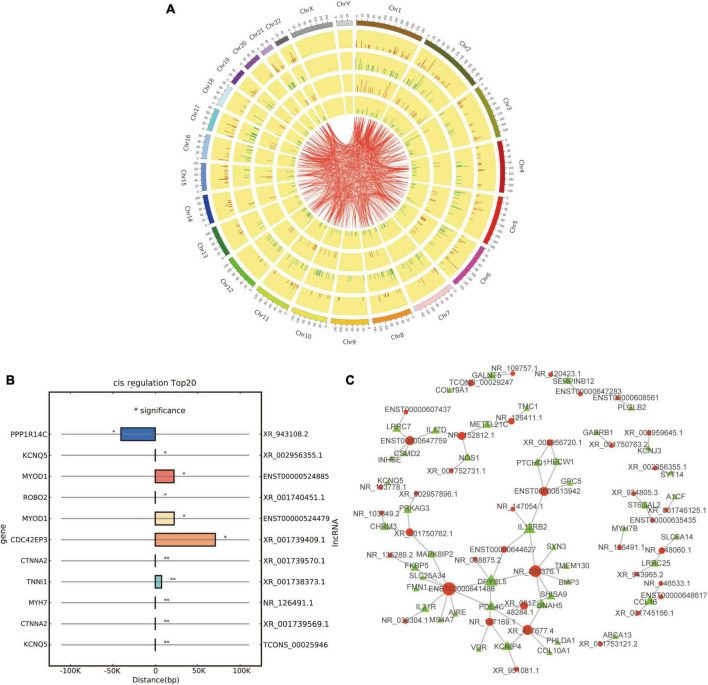
Co-expression and cis/*trans* regulation of differentially expressed lncRNAs. **(A)** Circos plot based on the co-expression analysis of differentially expressed lncRNAs and mRNAs; red and green lines represent up-regulation and down-regulation, respectively. **(B)** Top 20 potential target genes by *cis* regulation of differentially expressed lncRNAs; *p < 0.05 and **p < 0.01. **(C)** *Trans* regulation network constructed with 40 differentially expressed lncRNAs and their potential target genes; red and green dots represent lncRNAs and target genes, respectively.

Co-expression analysis was conducted between differentially expressed lncRNAs and mRNAs. As a result, a total of 15,454 pairs of lncRNA and mRNA were selected, such as *ENST00000414430* and *RGS1*, *ENST00000432120* and *CGA*, and *ENST00000601888* and *LGR5*. The distribution and relationship of the top 500 pairs of lncRNA and mRNA are displayed on a circos plot (Figure 6A).

Then, we tried to predict the target genes of differentially expressed lncRNAs by *cis* and *trans* regulation. As a result, the top 20 potential target genes by *cis* regulation were displayed, including *KCNQ5*, *CTNNA2*, and *MYH7* (Figure 6B). In addition, a lncRNA-target network was constructed using the top 40 related differentially expressed lncRNAs and their target genes by *trans* regulation (Figure 6C).

### 3.7 LncRNA-miRNA and lncRNA-TF-mRNA interactions

**FIGURE 7:**
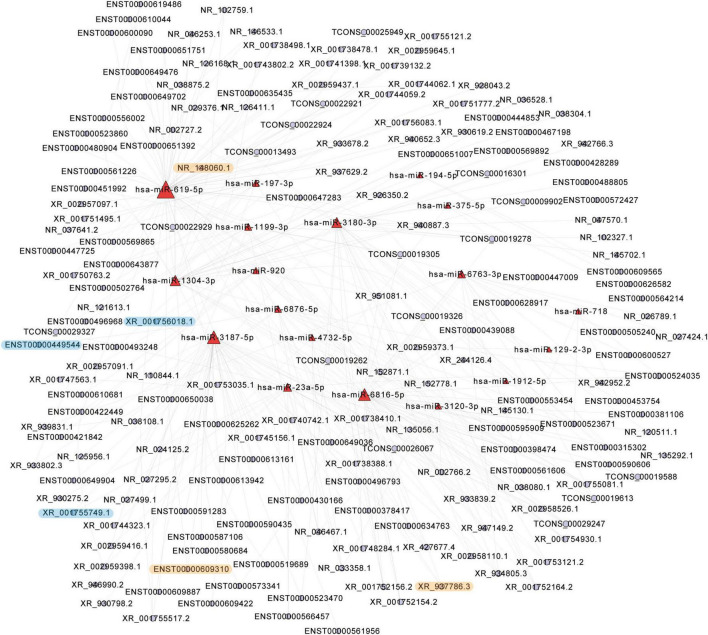
Top 300 lncRNA-micro RNA (miRNA) interaction network. Top 300 interaction pairs based on *p*-value of interaction of differentially expressed lncRNAs and differentially expressed miRNAs were shown. Red and blue dots represent miRNAs and lncRNAs, respectively. The significantly up-regulated lncRNAs were highlighted in yellow, and the significantly down-regulated lncRNAs were highlighted in blue.

To gain further insights into the roles of differentially expressed lncRNAs in the pathological alterations of EOMs of ONP, a lncRNA-miRNA interaction network was constructed. As a result, *hsa-miR-6816-5p* significantly interacted with 38 differentially expressed lncRNAs, such as up-regulated *ENST00000315302* and up-regulated *ENST00000381106* (*p* = 2.1E-07), and *hsa-miR-3120-3p* significantly interacted with six differentially expressed lncRNAs, such as down-regulated *ENST00000398474* and down-regulated *ENST00000523671* (*p* = 4.8E-07) (Figure 7).

**FIGURE 8:**
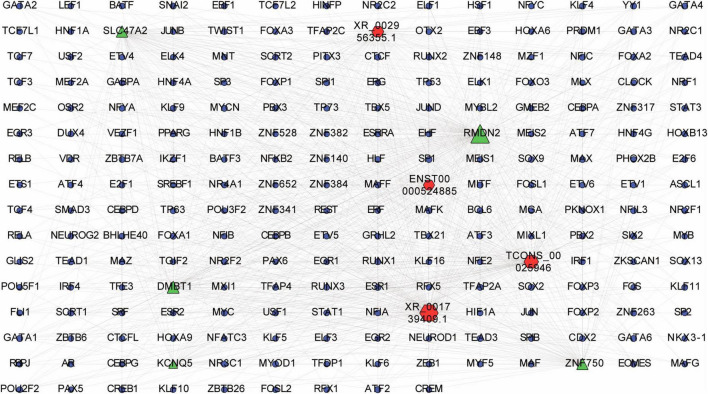
LncRNA-transcription factor (TF)-mRNA interaction network. Interaction network was constructed among top 20 differentially expressed lncRNAs and their top 20 *cis-*regulated differentially expressed mRNAs. Red, green and blue dots represent lncRNAs, mRNAs and TFs, respectively.

According to the lncRNA-TF-mRNA network, up-regulated lncRNA *ENST00000524885* and its co-expressed up-regulated mRNA *ZNF750* were significantly correlated with 84 TFs (*p* = 3.4E-06), such as *CREB1*, *TCF3*, and *TCF4*. In addition, up-regulated lncRNA *XR_001739409.1* and its co-expressed up-regulated mRNA *RMDN* were significantly related to 190 TFs (*p* = 6.3E-06), including *ESRRA*, *TP73* and *NFE2* (Figure 8).

### 3.8 Verification by RT-qPCR

**FIGURE 9:**
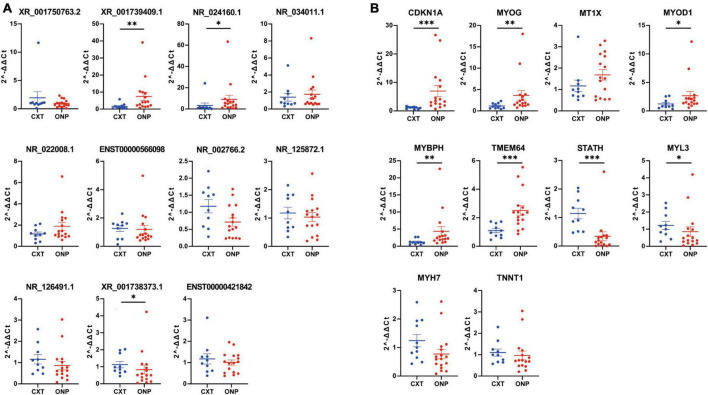
Expression levels of differentially expressed lncRNAs and mRNAs. Expression levels of **(A)** 11 differentially expressed lncRNAs (*XR_001750763.2*, *XR_001739409.1*, *NR_024160.1*, *NR_034011.1*, *NR_022008.1*, *ENST00000566098*, *NR_002766.2*, *NR_125872.1*, *NR_126491.1*, *XR_001738373.1*, and *ENST00000421842*) and **(B)** 10 differentially expressed mRNAs (*CDKN1A*, *MYOG*, *MT1X*, *MYOD1*, *MYBPH*, *TMEM64*, *STATH*, *MYL3*, *MYH7*, and *TNNT1*) were validated by RT-qPCR. *p < 0.05, **p < 0.01 and ***p < 0.001.

The expression levels of 11 differentially expressed lncRNAs (*XR_001750763.2*, *XR_001739409.1*, *NR_024160.1*, *NR_034011.1*, *NR_022008.1*, *ENST00000566098*, *NR_002766.2*, *NR_125872.1*, *NR_126491.1*, *XR_001738373.1*, and *ENST00000421842*) were detected by RT-qPCR in medial rectus muscle tissue samples from ONP and CXT patients, as well as 10 differentially expressed mRNAs (*CDKN1A*, *MYOG*, *MT1X*, *MYOD1*, *MYBPH*, *TMEM64*, *STATH*, *MYL3*, *MYH7*, and *TNNT1*). As a result, the expression levels of up-regulated lncRNAs (*XR_001739409.1* and *NR_024160.1*) and up-regulated mRNAs (*CDKN1A*, *MYOG*, *MYOD1*, *MYBPH*, and *TMEM64*) were increased in ONP samples, while down-regulated lncRNA (*XR_001738373.1*) and down-regulated mRNAs (*STATH* and *MYL3*) showed decreased expression in the ONP samples (Figure 9).

Next, we used Spearman’s correlation test to assess the correlation between the duration of palsy and the expression levels of these lncRNAs and mRNAs based on RT-qPCR results. No significant correlation was found between the duration of palsy and the expression levels of these lncRNAs and mRNAs.

### 3.9 ROC curve analysis

**FIGURE 10:**
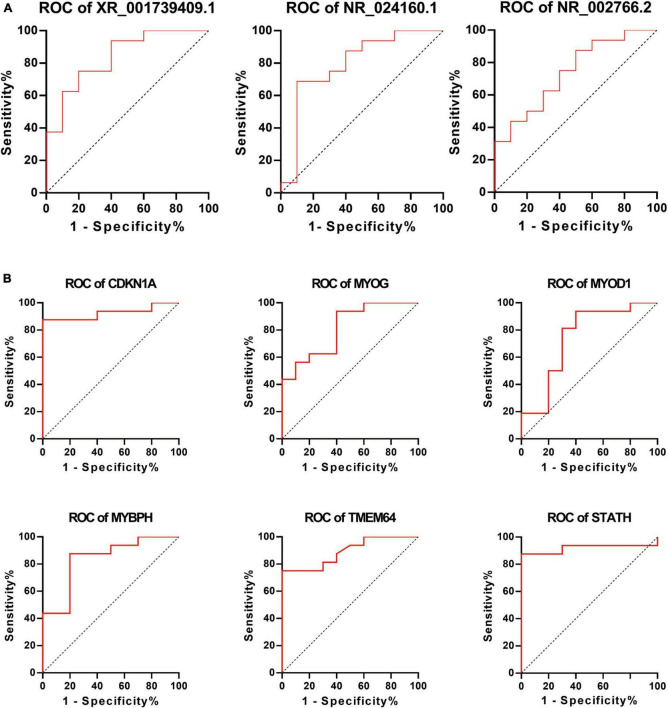
Receiver Operating Characteristic (ROC) curve analysis of differentially expressed lncRNAs and mRNAs. ROC curve shows the diagnostic performances of **(A)** lncRNAs (*XR_001739409.1*, *NR_024160.1*, and *NR_002766.2*) and **(B)** mRNAs (*CDKN1A*, *MYOG*, *MYOD1*, *MYBPH*, *TMEM64*, and *STATH*). The area under the curve (AUC) was **(A)** 0.8375, 0.7938, and 0.7375 and **(B)** 0.9250, 0.8125, 0.7438, 0.8375, 0.8906, and 0.9188, respectively.

To further elucidate the clinical importance of differentially expressed lncRNAs and mRNAs, the ROC curve was utilized to analyze these lncRNAs and mRNAs using sensitivity and specificity as targets for predicting and diagnosing ONP. Better sensitivity and specificity are indicated by a higher AUC (maximum AUC = 1). Differentially expressed lncRNAs including *XR_001739409.1*, *NR_024160.1*, and *NR_002766.2*, as well as mRNAs including *CDKN1A*, *MYOG*, *MYOD1*, *MYBPH*, *TMEM64*, and *STATH* had an AUC > 0.7 and a *p*-value < 0.05, suggesting their potential as biomarkers of ONP (Figure 10).

## 4 Discussion

Although various studies have focused on the etiology and treatment of ONP, studies on the EOM features of ONP, especially the molecular biology of EOMs, have been rather underexplored. In our study, medial rectus muscle tissue samples from four ONP patients and four CXT patients were collected for RNA sequencing. As a result, 33,515 predicted lncRNAs were mainly distributed on Chr1 and located on intergenic regions, with a length of more than 2,000 nt. In addition, a total of 618 differentially expressed lncRNAs (including 325 up-regulated and 293 down-regulated) and 322 differentially expressed mRNAs (including 120 up-regulated and 202 down-regulated) were identified between ONP and CXT groups.

Skeletal muscle innervation comprises stable and functional nerve interactions with muscles, which is critical for the maintenance of normal muscle structure and function (Kostrominova, 2022). Loss of motor innervation may induce rapid skeletal muscle fiber degeneration, causing muscle atrophy and fibrosis, which was verified using histopathological analysis in our study (Supplementary Figure 2).

Skeletal muscle atrophy is characterized by muscle alterations such as myofiber shrinkage, changes in fiber types or myosin isoforms, and net losses of cytoplasm, organelles, and total protein (Chemello et al., 2011). According to GO analysis (Figure 5B), down-regulated *MYH7* and *MYL3* were significantly correlated with the composition of muscle fibers, and down-regulated *MYH7* and *TNNT1* were involved in the contraction force of muscle fibers. The down-regulation of *MYH7*, *MYL3*, and *TNNT1* was validated by RT-qPCR, among which *MYL3* expression level was significantly decreased (Figure 9B). *MYH7* is the encoding gene of an isoform of the myosin heavy chain, which is known as a significant determinant of muscle shortening velocity and twitch kinetics (Park et al., 2012). The expression level of the *MYH7* protein has been found to be decreased in strabismic extraocular muscles compared with normal controls (Agarwal et al., 2016). *MYL3* encodes the myosin essential light chain of the sarcomere, which is important for structural stability of the α-helical lever arm domain of the myosin head (Hernandez et al., 2007). Mutations of *MYH7* and *MYL3* have been reported in various myopathies such as hypertrophic cardiomyopathy (Richard et al., 2003; Ingles et al., 2019). *TNNT1* is the encoding gene of slow skeletal muscle troponin T, which is a central player in the calcium regulation of actin thin filament function and is indispensable for the contraction of striated muscles (Wei and Jin, 2016). Mutations in *TNNT1* have been reported to result in nemaline myopathy (Jin et al., 2003). Thus, the decreased expressions of *MYH7*, *MYL3* and *TNNT1* might be associated with muscle atrophy and dysfunction caused by ONP.

Fibrosis is a common pathological change seen in denervated skeletal muscle, caused by excessive accumulation of extracellular matrix that replaces functional tissue (Rebolledo et al., 2019). According to GO analysis (Figure 5A), up-regulated mRNAs such as *CHI3L1* and *COL19A1* were significantly related to the components and metabolism of extracellular matrix. Both *CHI3L1* and *COL19A1* are the encoding genes of extracellular matrix proteins. *CHI3L1*, a glycoprotein with a molecular weight of 40 kDa, is found to be up-regulated in non-neoplastic diseases characterized by chronic inflammation and tissue remodeling (Coffman, 2008). In human skeletal muscle cells, *CHI3L1* can mitigate TNFα-mediated inflammation and insulin resistance (Görgens et al., 2014). *COL19A1* is the encoding gene of collagen type XIX, which is closely linked to the basement membrane zone in different tissues (Calvo et al., 2020). Thus, the up-regulation of *CHI3L1* and *COL19A1* might be associated with excessive accumulation of extracellular matrix in fibrotic EOMs of ONP. To sum up, the alternations in expressions of the above genes showed strong relation to muscle atrophy and fibrosis in EOMs of ONP, which is consistent with our findings in histopathologic analysis (Supplementary Figure 2).

In addition, up-regulated mRNAs such as *CHRM3* and *CHRND* were correlated with cholinergic synaptic transmission according to GO analysis (Figure 5A). Both *CHRM3* and *CHRND* are the encoding genes of acetylcholine receptor, which is a crucial component responsible for synaptic transmission at the neuromuscular junction. *CHRM3* was reported to enhance glucose uptake in L6 skeletal muscle cells by an AMPK-dependent mechanism (Merlin et al., 2010). Mutations of *CHRND* gene may cause congenital myasthenic syndrome by impairing co-clustering of the acetylcholine receptor with rapsyn (Müller et al., 2006). In our study, we considered that the up-regulation of *CHRM3* and *CHRND* might be a compensatory response related to denervation of EOMs in ONP.

Among the differentially expressed genes, the expression levels of *CDKN1A* (FC = 4.59), *MYOG* (FC = 2.86) and *MYOD1* (FC = 2.24) were the most significantly increased in the EOMs of ONP, as validated by RT-qPCR (Figure 9B). According to previous studies, the encoding protein of *CDKN1A*, called p21, is the founding member of cyclin-dependent kinase inhibitors, which participates in a wide range of processes such as cell cycle regulation, differentiation, cell migration (Kreis et al., 2019). In skeletal muscle, *CDKN1A* can be targeted by miR-208b and promote skeletal muscle cell proliferation (Wang et al., 2019). Thus, the up-regulation of *CDKN1A* might play a regulatory role in muscle cell proliferation in the EOMs of ONP. *MYOG* and *MYOD1* both participate in the composition of the myogenic bHLH family of transcription factors (Pownall et al., 2002). *MYOG* is an essential regulator of muscle stem cell homeostasis and adult myofiber growth (Ganassi et al., 2020), whereas *MYOD1* functions as a nodal point during specification of the muscle cell lineage (Weintraub et al., 1991). The expression level of *MYOG* has been found to be increased in the paralytic lateral rectus muscle compared with those with concomitant esotropia (controls) (Xia et al., 2021). Moreover, a study by Zhu et al. (2013) found that the expression levels of both *MYOG* and *MYOD1* were reduced in the extraocular muscles of 18 patients with concomitant strabismus compared with normal controls. Our study has revealed the up-regulation of *MYOG* and *MYOD1* in EOMs of ONP, which might be a feedback to muscle atrophy and dysfunction.

The regulatory role of lncRNAs in genomic and epigenomic has been gradually revealed. By interacting with the nucleic acids and proteins, lncRNAs can regulate gene expression in the nucleus and cytoplasm at both transcriptional and post-transcriptional levels (Herman et al., 2022). Especially, some lncRNAs may act as local regulators by recruiting regulatory complexes through RNA-protein interactions to influence the expression of nearby genes (Engreitz et al., 2016). Therefore, these lncRNAs are often correlated with the expression of nearby genes.

In this study, only 23 genes *cis-*regulated by 28 differentially expressed lncRNAs were differentially expressed (Figure 4), indicating that lncRNA regulation may only play a role in a few differentially expressed genes in the EOMs of ONP. However, two pairs of dysregulated lncRNA-*cis* gene showed strong interaction according to co-expression analysis and target gene prediction (Figure 6), which attracted our attention. Down-regulated *MYH7* was positively co-expressed with and *cis-*regulated by down-regulated *NR_126491.1* (*p* = 6.5E-05), and up-regulated *MYOD1* was positively co-expressed with and *cis-*regulated by up-regulated *ENST00000524479* (*p* = 0.003). Based on the lncRNA-TF-mRNA interaction network (Figure 8), *NR_126491.1* and *MYH7* were significantly correlated with 37 TFs, among which *RUNX1* was significantly up-regulated. Thus, the positive correlation between *NR_126491.1* and *MYH7* as well as *ENST00000524479* and *MYOD1* indicated that they might play a regulatory role in the pathological changes of EOMs in ONP.

In clinical practice, even with supramaximal recess–resect procedures on the horizontal muscles for the treatment of ONP, a postoperative exo-drift is generally unavoidable (Saxena et al., 2009), and one of the main reasons for this is the paralysis of the medial rectus. In the current study, ROC curve analysis showed that lncRNAs (*XR_001739409.1*, *NR_024160.1* and *NR_002766.2*) and mRNAs (*CDKN1A*, *MYOG*, *MYOD1*, *MYBPH*, *TMEM64*, and *STATH*) were significantly correlated with the paralysis of medial rectus muscle (Figure 10), which indicated that these lncRNAs and mRNAs might play crucial roles in the EOMs of ONP, suggesting their potential as biomarkers of ONP as well.

However, there remain discernible limitations in our research. For instance, the control group in our study is the EOMs from CXT patients rather than healthy controls. Furthermore, further investigations are required to delve into the intricate mechanisms underlying the molecular alterations observed in the EOMs of ONP.

In summary, our results provide a lncRNA and mRNA expression profile in medial rectus muscle of ONP. A total of 618 differentially expressed lncRNAs and 322 differentially expressed mRNAs were identified between the medial rectus muscle tissue samples from ONP and CXT patients. Functional enrichment analysis showed that mRNAs correlated with cholinergic synaptic transmission and the components and metabolism of extracellular matrix were significantly up-regulated, whereas down-regulated mRNAs were significantly correlated with the composition and contraction force of muscle fibers. Besides, down-regulated mRNA *MYH7* was *cis-*regulated and positively co-expressed with down-regulated lncRNA *NR_126491.1*, and up-regulated mRNA *MYOD1* was *cis-*regulated and positively co-expressed with up-regulated lncRNA *ENST00000524479*, suggesting their possible effect on the pathophysiological changes of EOMs of ONP. Moreover, ROC curve analysis suggested the important roles of lncRNAs (*XR_001739409.1*, *NR_024160.1*, and *NR_002766.2*) and mRNAs (*CDKN1A*, *MYOG*, *MYOD1*, *MYBPH*, *TMEM64*, and *STATH*) as biomarkers of ONP. These findings have provided novel insights into the molecular biology of EOMs of ONP, which may act as a reference for further studies or clinical practice.

## Data availability statement

The datasets presented in this study can be found in online repositories. The names of the repository/repositories and accession number(s) can be found in the article/Supplementary material.

## Ethics statement

The studies involving humans were approved by the Institutional Ethical Review Board of the Eye and ENT Hospital (2020070). The studies were conducted in accordance with the local legislation and institutional requirements. Written informed consent for participation in this study was provided by the participants’ legal guardians/next of kin.

## Author contributions

LW: Writing—original draft, Writing—review and editing, Conceptualization, Investigation, Software. MS: Writing—original draft, Writing—review and editing, Formal analysis, Investigation, Software. YL: Investigation, Software, Writing—review and editing. JH: Data curation, Methodology, Writing—review and editing. WX: Formal analysis, Visualization, Writing—review and editing. HB: Formal analysis, Visualization, Writing—review and editing. QZ: Writing—review and editing. CZ: Funding acquisition, Project administration, Resources, Writing—review and editing.

## Footnotes

1
http://geneontology.org/

2
https://www.kegg.jp/

3
https://CRAN.R-project.org/package=network

4
https://gtrd20-06.biouml.org/
