# Supplementary material for: The profiles and clinical significance of extraocular muscle-expressed lncRNAs and mRNAs in oculomotor nerve palsy
Source: Front Mol Neurosci. 2023 Dec 20;16:1293344. doi: 10.3389/fnmol.2023.1293344 (PMC10761543; doi:10.3389/fnmol.2023.1293344)
Supplement: Supplementary file 1 [file Data_Sheet_1.PDF]

Supplementary Material

Supplementary Figure 1. Sanger sequencing of TUBB3 sequences in congenital ONP cases.

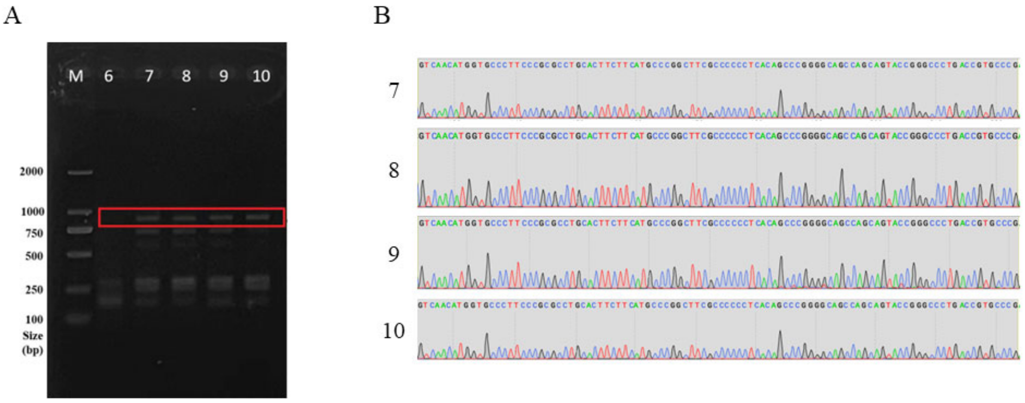

(A) The amplification product of TUBB3 detected by DNA electrophoresis. Product length: 836 bp. (B) The sequences of TUBB3 from c.763G to c.862G verified by Sanger sequencing. Case No. 6 was unable to undergo Sanger sequencing due to a low amount of amplification product.

Supplementary Figure 2. Histopathologic analysis of medial rectus muscle samples from ONP and CXT patients.

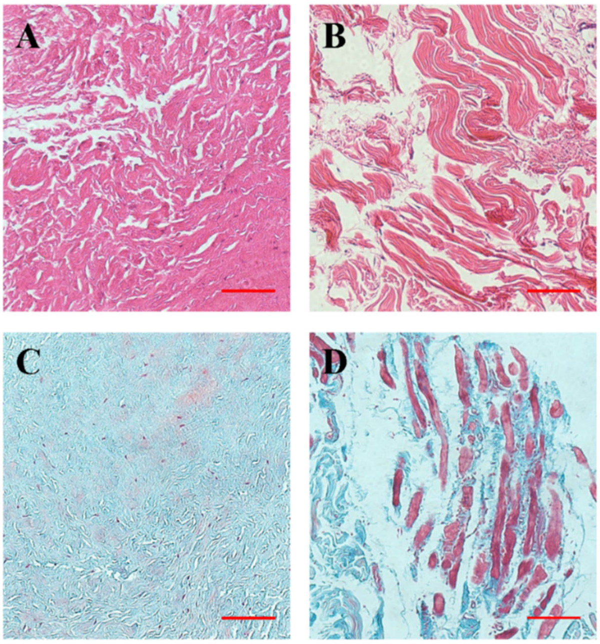

(A-B) HE staining of medial rectus muscle samples from ONP (A) and CXT (B) patients. (C-D) Masson staining of medial rectus muscle samples from ONP (C) and CXT (D) patients. Scare bar: 20 μm. Magnification as 200×.
